# Higher Plasma S100B Concentrations in Schizophrenia Patients, and Dependently Associated with Inflammatory Markers

**DOI:** 10.1038/srep27584

**Published:** 2016-06-09

**Authors:** Wu Hong, Min Zhao, Haozhe Li, Fanglan Peng, Fan Wang, Ningning Li, Hui Xiang, Yousong Su, Yueqi Huang, Shengyu Zhang, Guoqin Zhao, Rubai Zhou, Ling Mao, Zhiguang Lin, Yiru Fang, Qinting Zhang, Bin Xie

**Affiliations:** 1 Division of Mood Disorders, Shanghai Mental Health Center, Shanghai Jiao Tong University School of Medicine, Shanghai, 200030, China; 2 Shanghai Mental Health Center, Shanghai Jiao Tong University School of Medicine, Shanghai, 200030, China; 3 Shanghai Key Laboratory of Forensic Medicine, Institute of Forensic Science, Ministry of Justice, Shanghai, 200063, China; 4 XuHui District Mental Health Center of Shanghai, Shanghai, 200232, China

## Abstract

Glial damage and immune dysfunction are involved in pathogenesis of schizophrenia. However, interaction between glial damage and immune dysfunction in schizophrenia is undefined. This study aims to compare plasma S100 calcium binding protein (S100B) levels between schizophrenia patients and healthy participants, and to determine if immune markers are independently related with concentration of S100B in schizophrenia patients. Forty-one schizophrenia patients and thirty-three healthy volunteers were enrolled. Enzyme-linked immunosorbent assay (ELISA) was used to assess the concentrations of plasma S100B and inflammatory markers. We found that concentrations of S100B were elevated in schizophrenia patients than healthy participants (*p* < 0.05), and were negatively related with the severity of symptoms (*p* = 0.046). Receiver operating characteristic (ROC) curve analysis showed that different S100B levels between schizophrenia and healthy participants can be used as a clinical diagnostic factor (predictive value: 0.666, p = 0.015). Multiple linear regression analysis found that length of illness (Beta = −0.161), plasma levels of inflammatory regulation factors (including TGF-β1, logIL-23 and logIL-10) (Beta = 0.119, 0.475, 0.514) were independently associated with concentrations of S100B (Adjusted R^2^ = 0.897, *p* < 0.001). Therefore, our results suggest the possible function of S100B in pathogenesis of schizophrenia, and implicate the important role of autoimmune response and balance to glial dysfunction in patients with schizophrenia.

Schizophrenia is a severe mental illness with variety of symptoms that affects cognitive function, perceptual experiences, speaking and activities. Schizophrenia has become a severe public health problem and exerts enormous economic and personal costs worldwide.

Despite the ongoing tireless research efforts, the etiology of schizophrenia is still not clearly understood. Multiple hypotheses such as neurodevelopment hypothesis^1^, dopamine hypothesis^2^, glutamate hypothesis^3^, glial damage hypothesis^4^,^5^ and immune dysfunction hypothesis^6^,^7^, had been proposed.

During the past two decades, the associations between brain tissue damage, or glial cell dysfunction (astrocytes and oligodendrocytes) and schizophrenia have been repeatedly reported^4^,^5^. S100 calcium binding protein (S100B) proposed as a marker for glial dysfunction^8^ and blood-brain barrier disruption^9^, has been found increased in serum of patients with schizophrenia by numerous previous studies^4^,^10^,^11^,^12^. But, plasma concentrations of S100B were inconsistently reported in schizophrenia patients^13^,^14^,^15^,^16^,^17^. For instance, Gattaz *et al.* found decreased S100B plasma levels in schizophrenic patients, but Rothermundt *et al.* reported increased S100B plasma levels in unmedicated and treated schizophrenic patients^14^,^15^.

And, the correlations between S100B and schizophrenia subtypes, clinical characteristics were inconclusive too^14^,^15^,^17^,^18^,^19^. For instance, Rothermundt *et al.* found that levels of S100B were positively correlated with negative symptomatology, but Schmitt *et al.* found that levels of S100B were negatively correlated with deficit symptoms^15^,^20^, and some studies found no correlation^14^.

Similarly, during these decades, numerous studies from different areas showed that immune dysfunction was related with central neural system, and involved in the pathogenesis of schizophrenia^21^. Comprehensive studies have shown that schizophrenia patients had significant inflammatory markers alterations, such as Interleukin (IL)-1β, tumor necrosis factor-alpha, IL-6, IL-2, and transforming growth factor-beta, compared to healthy controls^22^. And, anti-inflammatory drugs like COX-2 inhibitors^23^, anti-TNF^24^, aspirin^25^ could improve the symptoms of schizophrenia patients^26^. In addition, animal studies also indicated that cytokines could lead schizophrenia-like behavior in animals^27^.

Among the hypotheses of schizophrenia, it was reported that inflammation can alter neurotransmitter^28^, neurodevelopment^29^, neurodegeneration^30^, and neural network activities and thus can induce psychiatric symptoms potentially. Studies have found that some antipsychotics arise the efficacy through inhibition of cytokine-mediated microglial activity^31^. For instance, Seki found that aripiprazole, an atypical antipsychotic, suppresses the TNF-α secretion from interferon-γ activated microglia and inhibits the apoptosis of rodent oligodendrocytes by interferon-γ activated microglia^32^.

Furthermore, microglial cells were demonstrated to be major immunocompetent cells of the brain and play an important role in the regulation of neuronal proliferation and differentiation. Pro-inflammatory cytokines could activate the microglial cells and induce the production of S100B, which potentially could injure neurons. Whereas, anti-inflammatory cytokines are beneficial for repairing damaged neuronal tissues^33^. Thus, inflammatory processes are linked to S100B, and play role of neurotoxicity in the brain.

With these facts in mind, we hypothesized that inflammatory markers are independently associated with the concentrations of S100B in schizophrenia patients. This study intend to compare the plasma concentrations of S100B between patients with schizophrenia and healthy volunteers, and to explore if the levels of inflammatory markers (including hsCRP, IL-17), regulation factors (including transforming growth factor-beta 1, IL-23, IL-10) and complement factor 3, are associated with plasma levels of S100B in patients with schizophrenia.

## Material and Methods

### Participants

In this study, forty one patients hospitalized in Shanghai Mental Health Center during 2014 were recruited, who were diagnosed schizophrenia according to the International Classification of Diseases-tenth edition (ICD-10) diagnostic criteria. Diagnosis and interviews were carried out by a trained clinical psychiatrist by semi structured clinical interview and review of medical records. Inclusion criteria were: (i) Age between 18 to 65 years old; (ii) Positive and Negative Symptom Scale (PANSS) total scores ≥60; (iii) Patients were drug naïve or drug free for at least 4 weeks before enrollment; (iv) Ability to read the research contents. Exclusion criteria were: (i)Alcohol and/or substance dependence or ever diagnosed with other psychiatric disorders; (ii) Pregnant or lactating; (iii) Physical diseases (cardiac disease, significant organic brain disease, diabetes mellitus, thyroid and other immune related disease, or other serious medical condition); (iv) Infectious, physical injury and autoimmune diseases, using anti-inflammatory drugs, corticosteroids or antibiotics in the recent four weeks; (v) Infectious and autoimmune diseases one week after enrollment; (vi) Lack of consensus on the diagnosis.

Thirty-three healthy volunteers, with gender and age matched, were invited to participate into the control group, who were staff members and medical students in Shanghai Mental Health Center. All the healthy controls were volunteers and met the following criteria. Inclusion criteria were: (i) Age between 18 to 65 years old; (ii) Ability to read the research contents. Exclusion criteria were: Family history of psychiatric disease, and some of the exclusion criteria of patients: from ii to v.

This study was approved by the Ethical Committee of Shanghai Mental Health Center, Shanghai, China. All the methods and procedures of this study were carried out in accordance with regulations and guidelines established by this committee. Informed and written consent were gained from all participants.

### Illness Severity and Clinical Variables Assessment

The symptoms and severity of patients were assessed using PANSS^34^. PANSS include three subscales (positive, negative and general) and three complemented scales. The positive symptoms were composed of seven symptoms, the negative symptoms were composed of seven symptoms, and the general symptoms were composed of 16 symptoms.

### Blood Sample Procedure

Blood samples were drawn on admission at about 8 a.m. from all subjects who were included in our study by venipuncture into a vacuum tube with Ethylene Diamine Tetraacetic Acid (EDTA). Samples were collected and centrifuged at 3000 g rates for 15 min at 4 °C, and plasma was obtained and stored at −70 °C until completion of the samples. Enzyme-linked immunosorbent assay (ELISA) kits were used to determine the plasma levels of S100B and inflammatory markers. The values of intra- and inter-assay coefficients were around 5%. Concentration is expressed as pg/ml (S100B, hsCRP, IL-23, IL-17 and IL-10), μg/ml (C3) and ng/ml (TGF-β1).

### Statistical Analysis

Data was analyzed using IBM Statistical Product and Service Solutions version 19.0 (IBM SPSS 19.0). Kolmogorov-Smirnov test was used for assessing normality of the variables distributions. Variables which were not normally distributed were log-transformed to be normally distributed. Data were presented as mean ± standard deviation (SD) and as median and inter-quartile range for normal distributed variables and non-normal distributed variables, respectively. The distribution of categorical variables was compared with two groups using chi-square tests.

Difference of normally distributed variables between the two groups was compared by *t* test. Binary logistic regression was used to measure the effect of significant variables that had been tested by univariate analyses among the patients with schizophrenia. Diagnostic accuracy of the plasma S100B was assessed by Receiver Operating Characteristic (ROC) curve analysis. Pearson's correlation coefficient was used to evaluate possible relationships between logS100B and PANSS scores (including all subscale scores and total score). Associations between predictor variables and level of plasma S100B were detected by multiple linear regression models. Only non-missing data were included in the modeling exercise. All statistical tests were two tailed and the criterion for significance level was set at *p* < 0.05.

## Results

### Participants Description

41 patients and 33 controls were enrolled in our study. Demographic and clinical data at base line of the two groups are showed in Table 1. As seen in Table 1, no significant difference between the two groups was observed in the demographic characteristic, regarding sex and age. The mean total PANSS score of patients was 80.29 (±9.220) and the mean sub-scale score was 23.05 (±6.20), 19.80 (±6.25), 37.44 (±5.91), 3.24 (±11.05), 9.00 (±3.60), 13.39 (±3.29), 6.17 (±2.34), 9.76 (±3.87), 8.12 (±3.62) 15.83(±4.91) respectively, for positive factors, negative factors, general factors, composite factors, lack of response, thought disorder, activation, paranoid, depression and attack dangerous.

### S100B in Patients with Schizophrenia and Healthy Volunteers

**Figure 1:**
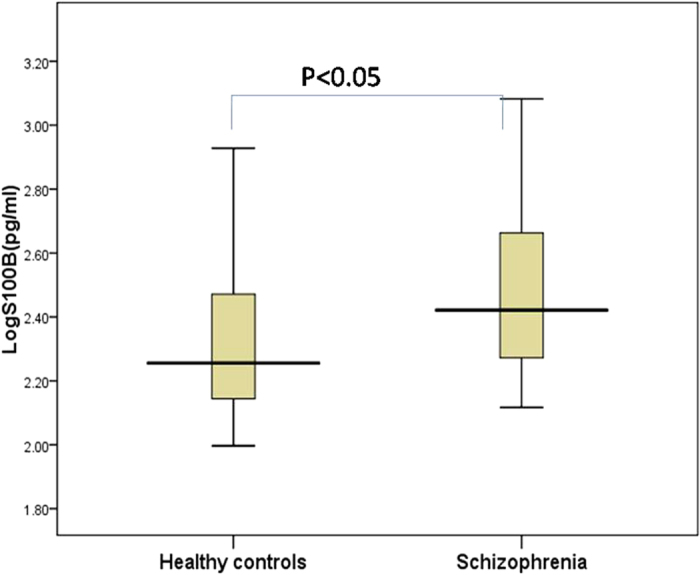
Levels of plasma levels of logS100B in patients diagnosed with schizophrenia (n = 41) and healthy control subjects (n = 33). Box plots showed higher levels of plasma logS100B (*t* = −2.152, *p* < 0.05) in patients with schizophrenia as compared to healthy controls. Statistical analysis was performed using *t* test.

S100B values were log-transformed to normalize data because of their abnormal. Patients with schizophrenia had significantly elevated plasma concentrations of logS100B (*t* = −2.152, *P* = 0.035) (Table 1, Fig. 1). The Binary logistic regression test after adjusting to age, sex, education level, marital status showed there is a direct effect of logS100B with schizophrenia (P = 0.019, odds ratio = 10.599; 95% confidence interval = 1.477–76.049).

**Figure 2:**
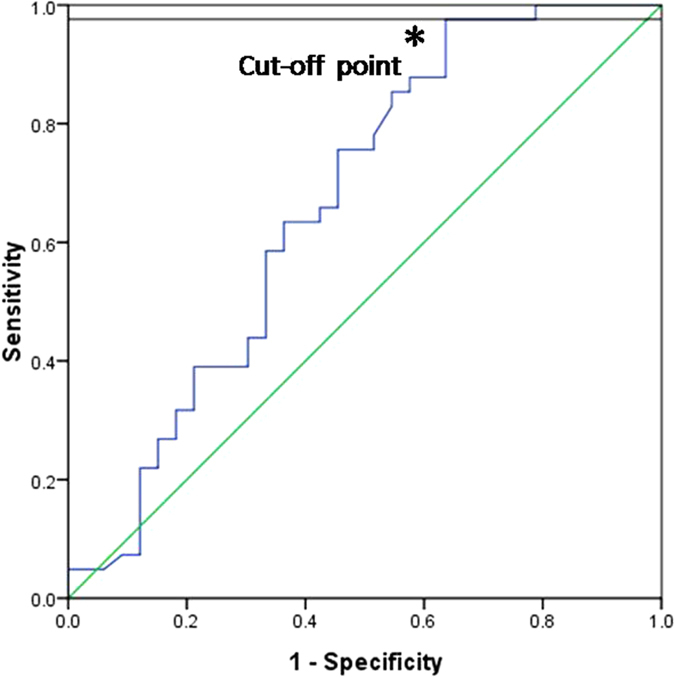
The receiver-operator characteristic (ROC) curve for level of plasma S100B in schizophrenia patients and healthy controls (AUC, area under the curve). The area under the curve (AUC) was 0.666 (95% confidence interval, 0.536 to 0.795; *p* = 0.015). When using 144.46 pg/ml as the cut-off value for the plasma S100B concentration, the sensitivity and specificity of S100B for the diagnosis of schizophrenia were 97.6% and 36.4%, respectively.

The ROC curves analysis revealed that the area under the curve (AUC) was 0.666 (95% confidence interval = 0.536–0.795; *p* = 0.015) (Fig. 2), when assessment the diagnostic accuracy of the plasma S100B concentration of comparison between patients with schizophrenia and healthy controls. The sensitivity and specificity of S100B for the diagnosis of schizophrenia were 97.6% and 36.4%, respectively, when using 144.46 pg/ml as the cut-off value for the plasma S100B concentration (Fig. 2).

### S100B Markers and PANSS Scores

**Figure 3:**
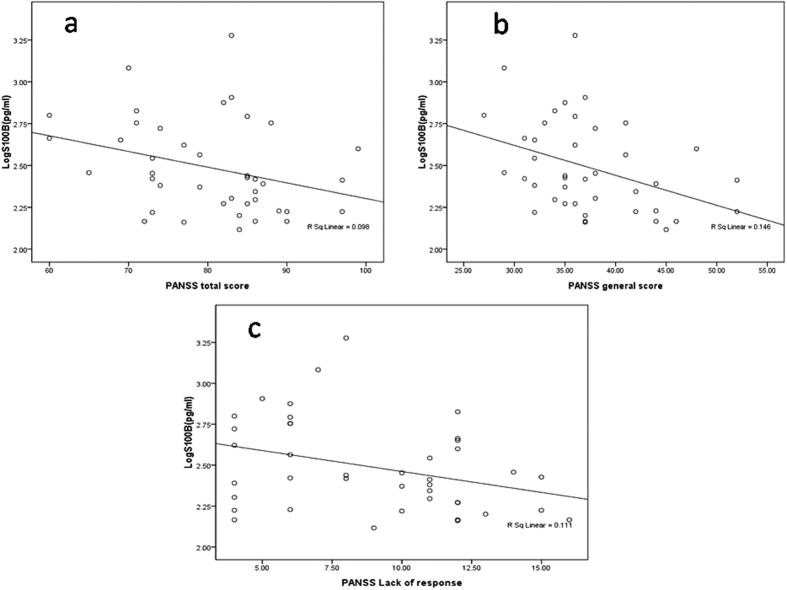
Correlation between S100B and PANSS score. Correlation plots show negative correlation between logS100B and total score, general score and lack of response of PANSS (*r* = −0.313, *p* = 0.046; *r* = −0.383, *p* = 0.014; *r* = −0.333, *p* = 0.034).

Correlation between logS100B and clinical characters in patients group was analyzed. There were remarkable negative correlations between logS100B and total score(*r* = −0.383, *p* = 0.014), general score (*r* = −0.313, *p* = 0.046) and the lack of response(*r* = −0.333, *p* = 0.034) of PANSS (Fig. 3). And no significant correlation between logS100B and other subscale score of PANSS was found.

### Factors Independently Associated with Levels of S100B

Characteristics including gender, current age in years, education, job, marital status, age of first onset, length of illness, number of episodes, and inflammatory markers including hsCRP, C3, TGF-β1, IL-17, IL-23, IL-10 were selected to be the independents for linear regression analysis. Values of IL-23 and IL-10 which were non-normally distributed were log-transformed to normalize their distributions. Significant positive correlations were found between education (*p* < 0.05), level of IL-17 (*p* < 0.001), logIL-23(*p* < 0.001), logIL-10(*p* < 0.001) and plasma level of logS100B. However, after multivariate regression analysis, independently associations were found between length of illness (Beta = −0.161, *p* < 0.01), TGF-β1 (Beta = 0.119, *p* < 0.05), logIL-23(Beta = 0.475, *p* < 0.001), logIL-10 (Beta = 0.514, *p* < 0.001) with plasma levels of logS100B (Table 2). The multivariate model, including 41 patients with schizophrenia with no missing data, show that length of illness, level of TGF-β1, logIL-23 and logIL-10 explained the majority of variance in logS100B (Adjusted R^2^ = 0.897, *p* < 0.001).

## Discussion

The first finding of this study is that schizophrenia patients have high levels of plasma S100B. Our finding is consistent with the results of numerous previous studies, which consistently found increased concentrations of S100B in blood of patients with schizophrenia^4^,^10^,^11^,^35^,^36^, although some contravariant reports^14^,^37^. Peripheral levels of S100B were reported consistence to those of cerebrospinal fluid, and represented a useful peripheral biomarker for brain tissue damages or glial dysfunction. But, S100B plays dual role in glial and neuronal cells, nanomolar concentrations of extracellular S100B act as a growth differentiating factor, while micromolar concentrations induce apoptosis^38^. Neuro-imaging study demonstrated higher S100B levels have been observed in white matter tracts, particularly in oligodendrocytes of the human corpus callosum (CC)^5^. Studies of microaray indicated decreased glial cells in brains, and alteration in genes about oligodendrocytes and astrocytes in patients with schizophrenia^39^.

Therefore, our result of high level of plasma S100B in schizophrenia patients reinforces the glial dysfunction and brain damages hypothesis in the pathogenesis of schizophrenia. It is important to meassure the degree of glial damage in schizophrenia patients by peripheral markers that can reflect level of centrol neural system^10^.

However, alteration levels of S100B is not specific to schizophrenia, but link to most neurodegenerative diseases, such as Alzheimer’s disease, mood disorders^40^. From results of our ROC curve analysis, when 144.46 pg/ml was used as the cut off value of the level of S100B, we found high sensitivity (97.6%), but low respecity(36.4%) of plasma concentrations of S100B for the diagnosis of schizophrenia. Therefore, biomarker values of S100B is not specific to diagnosis of schizophrenia, but may be a potential factor to predict symptoms’ severity and degree of brain damage and glial dysfunction.

The most important result of this study is that the level of TGF-β1, IL-23 and IL-10 were independently associated with plasma levels of S100B in patents with schizophrenia. This finding of inflammatory markers independently associated with the marker for glial dysfunction in patients with schizophrenia, suggests that inflammtory response possibly leads or gravates the glial dysfunction in patients with schizophrenia. The result is coincided with that of other studies exploring the association between inflammation and centrol neural development.

Khairova^41^ reported that inflammatory markers (TNF-alpha and IL-1) played a dual effect in synaptic plasticity and neural plasticity. As such, levels of S100B were reported increased by pro-inflammatory cytokines^42^, such as correlating with hsCRP in subjects with acute ischaemic stroke^43^, and been considered as a component of neuroinflammartory response^44^. Furthermore, increased S100B was reported being involved in the imbalanced inflammattory response in subjects with major depressive disorder^45^ and schizophrenia^46^.

TGF-β1 and IL-23 are closely related to Th17 pathway which acts important mediators of autoimmune diseases^47^. Previous studies have shown that autoimmune dysfunction was common to patients with schizophrenia and their unaffected ralatives^48^, and the risk of first episodes of psychosis was increased after the diagnosis of an autoimmune disease^49^,^50^. The value of TGF-β1 and IL-23 for indicating prognosis in manic patients was also found in our previours study^51^.

TGF-β1 is secreted by cells, including astrocytes in the brain, which were reported increased in schizophrenia, and identified as state markers for relapse^52^,^53^. Association between TGF-β1 and glial dysfunction were also reported in patients with Alzheimer's disease^54^.

IL-23 secreted by macrophages and dendritic cells is reported to be potential initiators of Th17 pathway, and be involved in activation, proliferation, differentiation and maintenance of Th17 cells, which subsequently activates the generation of pro-inflammatory cytokines thus amplifying immune response. Increased level of IL-23 was reported in schizophrenia patients, and could be a peripheral biomarker estimating antipsychotic therapy^55^. Relation between IL-23 and glial dsyfunction was also reported in patients with multiple sclerosis^56^.

While, IL-10 is an important Th2-type cytokine synthesized in the brain including microglia and astrocyte^57^, and inhibits expression of other pro-inflammatory cytokines. Hence, IL-10 contributes to suppression the immune and inflammatory response, being an anti-inflammatory mediator^58^ in central nervous system, and maintain the balance between levels of pro- and anti-inflammatory cytokine in central nervous system. IL-10 was found be altered in patients with schizophrenia, with contradictory results^59^,^60^.

Combined with the correllation between plasma level of S100B and IL-17 we found, this study help to bridge the gap between glial damage and immune dysfunction, especially the autoimmune imbalance hypotheses of schizophrenia. Our study is one of few studies to simultaneously assess the relationship between inflammatory markers and glial damage marker in patients with schizophrenia.

There are some limitations that should be addressed to interpreting the results. First of all, the relatively small sample size of patients and control groups decreased the power of the study to detect difference. Secondly, the patients were drug free at least for 4 weeks, however, long term effects of previous drugs on S100B and levels of inflammatory markers should be considered. Thirdly, plasma levels are disturbed by several confounding factors, but only some of those factors were considered in this study. Lastly, we did not assess the S100B and inflammatory markers concentrations in the patients with other severe mental illness, such as major depressive disorder and bipolar disorders. Therefore, further studies about S100B and inflammatory markers in schizophrenia and other psychiatric disorders from large-scale populations and strictly control of confounding variables are necessary to elucidate the role of S100B and inflammatory markers in schizophrenia, and values of biomarker.

In conclusion, findings of our study demonstrate high levels of plasma S100B in patients with schizophrenia. Levels of plasma autoimmune and anti-inflammatory markers are associated with the levels of S100B in patients with schizophrenia. These results suggest a possible role of S100B in the pathogenesis of schizophrenia, and help to bridge the gap between autoimmune dysfunction and glial damage hypotheses of schizophrenia.

## Additional Information

**How to cite this article**: Hong, W. *et al.* Higher Plasma S100B Concentrations in Schizophrenia Patients, and Dependently Associated with Inflammatory Markers. *Sci. Rep.* **6**, 27584; doi: 10.1038/srep27584 (2016).

## Figures and Tables

**Table 1:** Demographical and clinical features of the patients and controls.

Parameter	Patients with SZ (N = 41)	Healthy controls (N = 33)	*t*or*Z*or *X*^*2*^	*p*Value
Current age in years (mean ± SD)	37.00 ± 11.30	35.15 ± 13.72	−0.636^a^	0.527
Gender (female)	23 (56.10%)	23 (69.70%)	1.438^b^	0.335
Age of SZ onset in years (mean ± SD)	25.37 ± 9.56			
Length of illness in months (mean ± SD)	139.22 ± 107.14			
Number of episodes (mean ± SD)	3.32 ± 2.24			
PANSS score (mean ± SD)				
Total	80.29 ± 9.22			
Positive symptoms	23.05 ± 6.20			
Negative symptoms	19.80 ± 6.25			
General symptoms	37.44 ± 5.91			
Composite score	3.24 ± 11.05			
Lack of response	9.00 ± 3.60			
Thought disorder	13.39 ± 3.29			
Activation	6.17 ± 2.34			
Paranoid	9.76 ± 3.87			
Depression	8.12 ± 3.62			
Attack dangerous	15.83 ± 4.91			
S100B levels (pg/ml) (median, IQR)	263.71, 178.12–493.52	180.20, 135.95–303.10	−2.436^c^	0.015
LogS100B levels (pg/ml) (mean ± SD)	2.49 ± 0.28	2.35 ± 0.29	−2.152^a^	0.035
hsCRP levels (pg/ml) (mean ± SD)	4.44 ± 1.26	3.18 ± 1.79	−3.414^a^	0.001
C3 levels (μg/ml) (mean ± SD)	161.19 ± 108.79	164.20 ± 88.04	0.129^a^	0.898
TGF-β1 levels (ng/ml) (mean ± SD)	2.45 ± 2.14	3.16 ± 4.21	0.873^a^	0.388
IL-23 levels (pg/ml) (median, IQR)	16.95, 6.26–155.33	18.36, 9.60–146.42	−0.642^c^	0.521
IL-17 levels (pg/ml) (mean ± SD)	37.02 ± 18.10	31.18 ± 16.99	−1.418^a^	0.160
IL-10 levels (pg/ml) (median, IQR)	14.00, 12.17–22.18	23.40, 13.17–32.89	−2.463^c^	0.014

Abbreviations: SZ = Schizophrenia; N = Number; PANNS = Positive and Negative Symptom Scale; MOAS = Modified Overt Aggression Scale; SD = Standard deviation; IQR = Interquartile range; S100B = S100 calcium binding protein B; HsCRP = high sensitive C reactive protein; C3 = complements C3; TGF-β1 = Transforming growth factor-beta1; IL = Interleukin.

^a^*t* test.

^b^chi-square test

^c^Mann-Whitney U test.

**Table 2:** Multivariate relationships (logS100B as dependent variable) analyzed by linear regression.

Predictors	Unstandardized Coefficients		Standardized Coefficients	*t*	*p*	95% Confidence Interval for B	
	B	SEB	Beta			Lower Bound	Upper Bound
Length of illness	0.00	0.00	−0.16	−3.16	0.00	0.00	0.00
TGF-β1	0.02	0.01	0.12	2.28	0.03	0.00	0.03
logIL-23	0.16	0.03	0.48	6.01	0.00	0.11	0.22
logIL-10	0.73	0.12	0.51	6.39	0.00	0.50	0.97

Abbreviations: S100B = S100 calcium binding protein B; TGF-β1 = Transforming growth factor-beta1; IL = Interleukin.
